# Self-Care Needs a Clinical Safety Net: Integrating Community Pharmacists Into Primary Care

**DOI:** 10.7759/cureus.116115

**Published:** 2026-09-11

**Authors:** Esteban Zavaleta-Monestel, Jeaustin Mora-Jiménez, José A Castro Gamboa, Kevin Cruz Mora

**Affiliations:** 1 Pharmacy, Hospital Clínica Bíblica, San José, CRI; 2 Research, Hospital Clínica Bíblica, San José, CRI

**Keywords:** community pharmacy, health equity, patient safety, referral pathways, self-care

## Abstract

Self-care is an essential component of health and can increase autonomy, improve access to health information and treatment, and support more appropriate use of formal healthcare services. This editorial focuses on medicine-related self-care. For clarity, self-medication refers here to the selection and use of medicines without a prescription or direct clinician instruction, whereas pharmacist-supported self-care refers to structured pharmacist assessment, advice, safety-netting, and referral within the broader self-care pathway. However, the expansion of medicine-related self-care also creates an important clinical and policy challenge. When people are expected to recognize symptoms, select medicines, monitor response, and determine when further assessment is necessary without adequate professional support, diagnostic uncertainty, treatment risk, costs, and responsibility can shift from health systems to individuals. This concern is particularly relevant for people who rely on self-care because formal services are difficult to access or afford. Community pharmacists are well positioned to provide a clinical interface between individual self-care and formal healthcare because of their accessibility and expertise in medicines, but this role should complement rather than substitute for medical care. Accessibility alone, however, does not ensure safe, coordinated, or equitable care. Pharmacist-supported self-care should therefore be integrated into primary care pathways, operate within nationally defined scopes of practice, and be supported by minimum standards for structured assessment, identification of red flags, safety-netting, documentation, appropriate medicine use, referral, and continuity. Governance and financing should protect professional independence and ensure that assessment and referral are not conditional on purchasing a medicine. Evaluation should focus on safety, appropriateness, equity, affordability, patient experience, and clinical outcomes rather than consultation volume alone. Self-care can extend access and strengthen individual agency, but care may begin outside conventional clinical settings without health-system responsibility ending there.

## Editorial

Self-care is a routine and necessary component of healthcare. People make decisions every day about symptoms, medicines, prevention, monitoring, and whether professional care is required. WHO defines self-care as the ability of individuals, families, and communities to promote health, prevent disease, maintain health, and cope with illness, with or without the support of a health or care worker, while emphasizing that self-care complements rather than replaces health systems and health professionals [1]. For clarity, we use self-care as the broad umbrella term throughout this editorial. Within that umbrella, self-medication refers specifically to the selection and use of medicines without a prescription or direct clinician instruction, whereas pharmacist-supported self-care refers to a structured clinical interaction in which a pharmacist assesses whether self-care is appropriate and provides advice, safety-netting, or referral as indicated. We use the term minor-ailment service only when referring to a defined service model or to studies that use that terminology. These distinctions matter, because self-care is frequently presented as a means of increasing autonomy, improving convenience, bringing care closer to communities, and reducing unnecessary demand on health services. These objectives are legitimate. Many health problems can be managed safely without a physician consultation, and people should be supported rather than discouraged from taking an active role in their health.

But enthusiasm for self-care should not obscure the circumstances in which it occurs. In 2023, an estimated 4.6 billion people worldwide were not fully covered by essential health services, while 2.1 billion experienced financial hardship from out-of-pocket health spending in 2022 [2]. Self-care occurs across a spectrum of circumstances. For many people, it is an informed and preferred way to manage appropriate health needs; for others, its use may also be shaped by waiting times, geographical barriers, cost, fragmented services, or difficulty obtaining formal care. This distinction matters because supporting a person's informed choice to use self-care is different from leaving that person to manage clinical uncertainty because accessible alternatives are lacking. If health systems expand self-care without establishing reliable mechanisms for assessment, escalation, and continuity, responsibility can gradually move from healthcare institutions to patients and families, who may be expected not only to manage a symptom but also to determine whether it represents a benign condition, an adverse treatment effect, deterioration of a chronic illness, or an early manifestation of serious disease. For well-informed patients with reliable access to healthcare, these responsibilities may be manageable. For individuals facing financial hardship, limited health literacy, language barriers, or geographical isolation, the same model may create additional risk. Self-care, in other words, needs a clinical safety net.

Medicine-related self-care and the management of common symptoms illustrate how such a safety net could operate, and community pharmacists are well-positioned to provide it. Community pharmacies are often among the most accessible points of contact with healthcare professionals, commonly permitting direct access without an appointment, and pharmacists bring specific expertise in medicines to that encounter. This role is increasingly important as the boundary between professional care and self-care becomes less distinct: people obtain medicines without prescriptions, use digital health information, monitor symptoms at home, and increasingly participate in decisions that previously occurred almost exclusively within medical consultations. In this environment, the pharmacist can serve not simply as a supplier of medicines but as an accessible clinical checkpoint. A person seeking treatment for an apparently minor symptom is not necessarily presenting with a minor illness; similar presentations can arise from self-limiting conditions, adverse medicine effects, poorly controlled chronic disease, or serious illness at an early stage. The purpose of pharmacist involvement should therefore not be merely to facilitate access to a product. It should be to help determine whether self-care is appropriate at all.

Accessibility, though, does not guarantee quality, and it should not be used as a surrogate for it. The clinical value of a pharmacy consultation depends on what happens during the encounter, and a pharmacist cannot reliably determine whether self-care is appropriate without obtaining sufficient information: symptom duration and severity, previous episodes, treatments already attempted, age, pregnancy, comorbidities, allergies, concurrent medicines, and warning features suggesting the need for further evaluation. That requirement is organizational as well as clinical. Meaningful triage requires sufficient staffing and protected time for assessment, a consultation environment that permits privacy and confidential questioning, pharmacists trained to recognize red flags and atypical presentations, and clear scope boundaries with appropriate quality oversight. Without these conditions, expanding pharmacist-supported self-care can introduce the very risks it is intended to reduce, including inconsistent assessment, missed serious illness, inappropriate or prolonged non-prescription medicine use, and delayed referral. Pharmacist involvement is therefore most likely to add value when it functions as a structured assessment, safety-netting, and referral layer rather than simply extending access to medicines. The available evidence supports this emphasis on structure. An Australian cluster-randomized controlled trial involving 894 participants evaluated a pharmacist-led minor-ailment service incorporating agreed clinical pathways, pharmacist training, structured documentation, and communication systems developed with general practitioners; compared with usual care, the structured service improved appropriate referral, adherence to referral advice, appropriate non-prescription medicine recommendations, and symptom resolution or relief [3]. The importance of structure becomes even clearer when contrasted with less standardized practice. A systematic review of 30 observational studies examining community pharmacists' management of minor ailments in countries classified by the authors as developing countries found substantial variation in information gathering and counseling; the reviewed studies reported no use of specific protocols or evidence of documentation, and inappropriate antibiotic recommendations for acute diarrhea and cough or cold presentations were reported in 10 of the 25 studies that assessed antibiotic recommendations [4]. Antimicrobial stewardship is therefore a concrete safety function of structured pharmacist-supported self-care, particularly where antibiotics may be obtainable without a prescription. The assessment should establish not only whether a medicine is appropriate, but whether the presentation can reasonably remain within self-care or instead requires further diagnostic evaluation. In this setting, the pharmacist's role is as much about preventing inappropriate treatment and delayed diagnosis as it is about recommending therapy. These findings are not an argument against pharmacists managing common conditions. They point instead to a more important conclusion: expanding a professional role without defining the clinical standards governing that role does not guarantee better care. A health system should therefore not measure the success of pharmacist-supported self-care solely by the number of consultations performed or the number of people who did not subsequently attend a physician appointment. The content and quality of those consultations matter.

That principle has a direct implication for how a consultation should unfold: assessment must precede treatment. In many community pharmacy settings, the encounter begins with a request for a specific product-an analgesic, a cough preparation, an antacid, or a topical treatment. The safest response is not necessarily to supply what was requested or to recommend an alternative; the first question is whether medicine-related self-care is appropriate at all. A structured assessment should identify circumstances in which the requested treatment is unsuitable, and it should identify situations in which treatment should be delayed because the patient requires diagnostic assessment. This distinction also protects against inappropriate medicalization. High-quality pharmacist-supported self-care does not necessarily result in a medicine being supplied; appropriate care may just as well consist of reassurance, non-pharmacological advice, discontinuation of an unnecessary treatment, or referral. The absence of a medicine sale should not be interpreted as an absence of clinical value.

Even an appropriate initial decision contains uncertainty because many diseases evolve over time, and a condition that appears uncomplicated at first may subsequently fail to improve or develop features requiring professional assessment. For this reason, every pharmacist-supported self-care consultation should include safety-netting: patients should understand what course they can reasonably expect, how long improvement should take, which warning symptoms should prompt earlier assessment, and where they should seek help. Advice simply to see a doctor if it gets worse places considerable interpretive responsibility on the patient, who may have a very different understanding of what deterioration means or how long symptoms should persist before further evaluation becomes necessary. Effective safety-netting is more specific than that. For example, a person seeking self-care for an acute cough might initially have no features requiring escalation, but the safety-net should specify the condition-specific reassessment interval defined by the clinical pathway and make clear that rapidly worsening symptoms, marked breathlessness, systemic deterioration, or other features suggesting serious illness require earlier assessment rather than continued self-treatment. Persistence beyond that defined interval should prompt reassessment rather than repeated purchase of symptomatic medicines. Effective safety-netting establishes an expected trajectory and an action plan, so that patients know whether improvement is expected within hours, days, or a longer period, and whether continued self-treatment beyond a defined period is inappropriate. Done well, safety-netting transforms the consultation from a single transaction into a clinical decision that anticipates uncertainty.

Referral needs the same rigor because apparent simplicity here, too, can conceal fragmentation. Telling a patient to consult a physician is not necessarily an effective referral: a patient may not know where to go, may be unable to obtain an appointment, or may not understand the urgency of the recommendation, and the receiving professional may have no information about the pharmacy assessment already performed. A clinically integrated model requires more. Referral pathways should define the circumstances requiring escalation, the appropriate destination, and the urgency of assessment, since a patient with a non-urgent condition requiring diagnostic clarification may need routine primary care, whereas a patient with a potentially serious red flag may need same-day assessment. Where feasible and with the person's consent, relevant clinical information should accompany the referral. Integration should also operate in the opposite direction. Where permitted by applicable regulation, supported by appropriate patient consent, and feasible within the available infrastructure, pharmacists undertaking clinical assessment should have access to the information necessary for a safe recommendation, particularly current medicines, allergies, relevant diagnoses, and, when clinically pertinent and available, recent laboratory or organ-function data. Continuity therefore depends on bidirectional information exchange rather than solely on transmitting the pharmacy assessment onward. Closed-loop electronic referral and interoperable health records can support this exchange, but health systems should not wait for advanced digital infrastructure before implementing basic continuity mechanisms; standardized documentation, referral forms, and defined professional responsibilities can provide basic continuity mechanisms even in settings with limited technological resources. A referral is not complete simply because responsibility has been verbally transferred. Continuity requires mechanisms that reduce loss to follow-up and preserve clinically relevant information across care settings.

Making any of this work also means confronting the commercial context in which community pharmacy operates. Many pharmacists practice within businesses whose revenue depends substantially on product sales. This does not mean pharmacists cannot provide independent professional advice; they routinely balance commercial and professional responsibilities. But the structure creates a potential conflict that policymakers should acknowledge rather than ignore. A self-care consultation may result in a recommendation to purchase a medicine, but it may also appropriately result in no medicine being supplied at all, because non-pharmacological management is preferable, an existing medicine should be reviewed, or the patient should be referred elsewhere. Payment systems should recognize clinical work rather than transactions alone. Possible mechanisms include fee-for-service payment for the clinical consultation itself, public-payer reimbursement for defined minor-ailment services, or incorporation of pharmacist assessment into bundled or capitated primary-care arrangements. The specific mechanism will vary across health systems, but remuneration should attach to assessment and clinical decision-making rather than to whether a medicine is sold. Otherwise, referral, reassurance, or a decision not to supply a medicine can be systematically undervalued. This has a further, equity-related implication: assessment and referral should not depend on the patient's willingness or ability to purchase a product. If professional advice is effectively financed only through medicine sales, people who cannot afford treatment may be disadvantaged precisely at the point at which they need help navigating the health system. The pharmacist's clinical role should remain distinguishable from the commercial transaction.

Geographical accessibility is often cited in support of the equity potential of pharmacist-supported services delivered through community pharmacies, but it is only one dimension of equitable care. A pharmacy may be nearby while the recommended treatment remains unaffordable, or a patient may receive appropriate referral advice but have no realistic access to the service to which they are referred. Language, health literacy, cultural barriers, and financial constraints may all influence whether a person can actually benefit from pharmacist-supported self-care delivered through community pharmacies. Implementing pharmacist-supported self-care through community pharmacies should therefore not be presented automatically as an equity intervention; equity should be demonstrated rather than assumed. Health systems implementing pharmacist-supported self-care should ask who uses the service, who does not, whether referrals are completed, and whether outcomes differ among populations already experiencing barriers to healthcare, especially since the people who depend most heavily on accessible self-care may be those with the fewest alternatives. A poorly designed model could therefore have a paradoxical effect, improving convenience for people who already navigate healthcare effectively while providing a lower level of continuity for people unable to obtain formal services.

How pharmacist-supported self-care is evaluated will, in turn, shape how it is designed. Health systems frequently emphasize activity measures: the number of consultations performed, prescriptions avoided, or estimated cost savings. These outcomes are useful for planning, but they are insufficient indicators of quality. A systematic review of international community pharmacy literature identified six interrelated dimensions of quality: person-centered care, access, environment, safety, competence, and integration within local healthcare systems [5]. Consultation volume alone captures none of these dimensions adequately. A consultation should not automatically be considered successful because the patient did not subsequently visit a physician; avoiding an unnecessary appointment may represent efficient care, but failure to obtain necessary medical assessment may represent the opposite. Evaluation should therefore include whether medicines were appropriate, whether red flags were identified, whether referrals were clinically justified and followed, whether symptoms resolved as expected, and whether the service was affordable and acceptable. Patient experience and understanding matter as well, since a technically correct recommendation may still fail if the patient cannot afford it, cannot follow the proposed pathway, or misunderstands the purpose and limits of the pharmacist-supported service. The central evaluation question is not how many consultations were moved to pharmacies, but whether patients received safe, appropriate, coordinated, and equitable care.

Pharmacist-supported self-care should not be framed as a substitute for medical care. Its value lies in providing an additional point of entry into an integrated system, with clear boundaries between self-management, pharmacist assessment, and escalation to other clinicians. This requires a complementary rather than competitive relationship with physicians and other primary-care professionals. Pharmacists can assess and manage presentations that fall within their authorized scope and established pathways, whereas medical assessment remains essential when diagnostic uncertainty, red flags, treatment failure, complex comorbidity, or the need for examination, testing, or treatment beyond the pharmacist's scope makes continued self-care inappropriate. The strongest model is therefore one in which patients can enter through multiple accessible points while remaining connected to the level of care their clinical situation requires.

The regulatory framework is therefore integral to how pharmacist-supported self-care can operate. Regulation determines or constrains which medicines can be supplied without a prescription, which clinical activities pharmacists are authorized to perform, and the requirements governing documentation, referral, information sharing, and professional accountability. These arrangements differ across jurisdictions, so the model proposed here should not be interpreted as advocating a universal expansion of pharmacists' legal scope. Rather, assessment, red-flag recognition, safety-netting, documentation, and appropriate escalation represent core clinical functions that should be implemented within national law, professional regulation, and locally defined scopes of practice.

Public expectations are another important part of implementation, but they should not be assumed to be uniform across populations or health systems. Services should assess how patients understand the pharmacist's role, what they expect from a pharmacy consultation, and whether they recognize when referral to another clinician remains necessary. The objective should not be to encourage a perception of pharmacists as substitutes for physicians, but to make the boundaries and purpose of pharmacist-supported self-care understandable. Public and patient communication should therefore explain what the service can provide, what it cannot replace, when escalation may be recommended, and why a consultation may appropriately end without a medicine being supplied. Where expectations do not align with the locally authorized scope of practice, the appropriate response is to clarify those expectations rather than simply expand them.

Against this background, the central proposal of this Editorial is a minimum-function framework that can apply across health systems even when implementation differs. Pharmacist-supported self-care should involve trained pharmacists with sufficient time and privacy to perform a structured assessment and determine whether self-care is appropriate; consideration of red flags and relevant medicine-related risks; clear safety-netting advice, including expected duration and warning symptoms; defined referral thresholds and levels of urgency; documentation of clinically relevant consultations; communication with other healthcare professionals when necessary and feasible; and professional judgment protected from inappropriate commercial pressure, with assessment never conditional on purchasing a product. None of this requires every country to reproduce the same service model: a high-income health system with interoperable electronic records may implement these principles differently from a resource-constrained setting where paper documentation and telephone communication remain necessary. The purpose of minimum standards is not to impose identical infrastructure but to preserve the clinical functions necessary for safe care, and that distinction matters if pharmacist-supported self-care is to contribute meaningfully to universal health coverage. Coverage is not achieved simply because a health professional or medicine is physically accessible; it must also provide sufficient quality, continuity, and financial protection to improve health.

Self-care should be encouraged when it gives people greater control over their health, and pharmacists can make this model substantially stronger by bringing professional judgment closer to where self-care decisions occur. But self-care should not become a mechanism for moving risk out of formal healthcare. The challenge for health systems, then, is not to choose between self-care and professional care. It is to connect them. Community pharmacists can provide an important clinical interface between individual action and formal health services, but accessibility alone is insufficient; their contribution should be supported by structured assessment, safety-netting, defined referral pathways, professional independence, appropriate remuneration, and evaluation based on safety and equity. The success of pharmacist-supported self-care should ultimately be judged not by how many patients are kept away from clinics, but by whether people receive the right level of care at the right time. As increasing amounts of healthcare take place in homes, pharmacies, and communities, the physical boundaries of the clinic will matter less. Care may begin outside the clinic, but health system responsibility for helping people reach safe and appropriate care should not end there.
